# Midlife Hypertensive Status and Cognitive Function 20 Years Later: The Southall and Brent Revisited Study

**DOI:** 10.1111/jgs.12416

**Published:** 2013-08-26

**Authors:** Clare Taylor, Therese Tillin, Nish Chaturvedi, Michael Dewey, Cleusa P Ferri, Alun Hughes, Martin Prince, Marcus Richards, Ajit Shah, Robert Stewart

**Affiliations:** * King’s College London (Institute of Psychiatry)London, UK; † National Heart and Lung Institute, Imperial College LondonLondon, UK; ‡ Department of Psychiatry, Universidade Federal de São PauloSxão Paulo, Brazil; § Department of Psychobiology, Universidade Federal de São PauloSão Paulo, Brazil; ∥ Medical Research Council Study of Health and DevelopmentLondon, UK; # School of Health, University of Central LancashirePreston, UK

**Keywords:** cognitive impairment, hypertension, blood pressure, pulse pressure, ambulatory blood pressure

## Abstract

**Objectives:** To investigate long-term prospective associations between a range of measurements of hypertensive status in midlife and cognitive impairment 20 years later.

**Design:** Cohort study.

**Setting:** Two areas (Southall and Brent) of northwest London.

**Participants:** Survey samples of a multiethnic population (European, African Caribbean, South Asian) aged 40 to 67 were followed up 20 years later.

**Measurements:** Comprehensive cardiovascular assessments were performed at baseline, including measurements of resting blood pressure (BP) and, in a subsample, ambulatory BP. At follow-up, a battery of cognitive assessments was administered, and a composite outcome was derived, with impairment defined as the lowest 10% within each ethnic group. Logistic regression models were used to investigate associations with prior measures of hypertensive status.

**Results:** In 1,484 participants at follow-up, cognitive impairment showed significant U-shaped associations with baseline diastolic BP (DBP) and mean arterial pressure (MAP; strongest for those aged ≥50 at baseline), independent of a range of covariates, but no associations were found with systolic BP or pulse pressure. Cognitive impairment was also associated with antihypertensive medication use and higher evening ambulatory DBP at baseline. No substantial differences in strengths of association were found between ethnic groups.

**Conclusion:** Low and high DBP and MAP were associated with cognitive impairment 20 years later. Higher evening DBP on ambulatory monitoring was also associated with greater risk.

**C**ognitive impairment is a matter of increasing public health importance with rapid demographic aging. Hypertension is a risk factor for cognitive impairment and is important because of its high prevalence and potential for modification. Relationships between blood pressure (BP) levels or exposure to hypertension and cognitive impairment or dementia as outcomes have been reviewed extensively,^1^ and findings from cross-sectional studies and short-duration prospective studies have been heterogeneous, including associations with high BP, with low BP, and with high and low BP (U-shaped relationship). The few cohort studies with longer than a 10-year interval between BP measurement and cognitive examination have found that higher midlife BP is incrementally associated with greater risk of later dementia or cognitive impairment, although findings have been inconsistent in terms of the component of BP most associated with cognitive outcomes and the role of treated hypertension as a risk factor.^2^–^6^

Other measures of hypertension have received much less research. Pulse pressure (PP), a proxy measure of arterial stiffness associated with greater risk of cardiovascular events,^7^ has been found to be associated with dementia and cognitive decline or impairment when measured in late life,^8^–^10^ but few, if any, data have been reported on midlife PP or mean arterial pressure (MAP) in relation to later cognitive outcomes. Ambulatory BP is a better predictor of cardiovascular events and adverse outcomes than clinic BP measurement,^11^ and a recent study found this also to be true for 24-hour SBP in relation to white matter hyperintensity volume and cognitive decline as outcomes.^12^ Finally, urinary albumin excretion is an indicator of microvascular disease 13 but has received little attention as a possible mechanism for the effect of hypertension on cognitive function. The aim of this analysis was to investigate associations between comprehensive measures of midlife hypertension and later-life cognitive impairment.

## Methods

### Study Design and Participants

The Southall and Brent Revisited (SABRE) Study was designed to follow up cardiovascular outcomes in community samples of European, South Asian, and African-Caribbean residents who had originally been recruited in the London boroughs of Brent and Southall in 1988 to 1991. Baseline surveys had investigated cardiometabolic risk in residents aged 40 to 69 from three ethnic groups (European, n = 2,346; South Asian, n = 1,710; and African Caribbean, n = 801) and have been described previously.^14^ Participants were recruited from primary care lists and industrial workforces, with response rates ranging from 58% to 71%. Because of the nature of the recruitment waves,^14^ there was a male preponderance in the baseline sample. All South Asian and African-Caribbean participants were first-generation migrants, most of whom had moved to the United Kingdom in the 1950s and 1960s. Exclusion criteria were cancer and renal failure, severe disability, and severe psychiatric disturbance.

The SABRE follow-up study approached surviving participants still living in England or Wales. Participants were traced using the UK National Health Service tracing system in 2008, 20 years after baseline, and data collection took place between 2008 and 2011. Home assessments were attempted for consenting participants unable or unwilling to attend clinic. All participants gave written informed consent. Approval for the baseline study was obtained from Ealing, Hounslow and Spelthorne, Parkside, and University College London research ethics committees and at follow-up from St Mary’s Hospital Research Ethics Committee (ref. 07/H0712/109).

### Baseline Assessments

Participants were asked to complete a health and lifestyle questionnaire at baseline. Health examinations were conducted after an overnight fast and included blood samples taken after fasting and after a 75-g glucose load. Brachial BP was measured at rest according to a standard protocol using a random zero sphygmomanometer.^15^ Participants were seated for 5 minutes before measurement on the right arm. Systolic and diastolic BP (SBP and DBP) were recorded as the average of two readings with an interval of at least 1 minute. Hypertensive treatment was ascertained from the participant questionnaire. African-Caribbean and European participants recruited to the Brent study center were also considered for ambulatory BP monitoring. Every other participant whose clinic BP was less than 140/90 mmHg and who was not known to have hypertension and all of those whose clinic BP was 140/90 mmHg or greater were offered ambulatory monitoring (TM2420, Takeda, Tokyo, Japan) for 24 hours after screening. The monitors were programmed to measure BP every 15 minutes during the day (8 a.m. to 10 p.m.) and every half hour at night.

### Covariates

Cardiovascular disease was defined on the basis of major Q waves on electrocardiogram at baseline or self-reported coronary heart disease or stroke. Height and weight were measured,^15^ body mass index (BMI) was calculated, and obesity was defined as a BMI greater than 30.0 kg/m^2^. Fasting blood samples were taken and glucose and cholesterol levels assayed. Diabetes mellitus was ascertained from self-report and retrospective application of World Health Organization (WHO) 1999 criteria.^16^ Albumin excretion rate (AER) was established from timed overnight urine collections using immunoturbidimetry.^17^ Smoking status was ascertained by questionnaire and coded as never, current, or previous, and alcohol consumption was coded as less than weekly, one to two times per week, and daily or almost daily.

### Follow-Up Assessment

Trained staff conducted cognitive assessments that lasted approximately 30 minutes early on the day of clinic attendance or home visit to avoid fatigue. The test battery consisted largely of tests successfully used in previous cross-cultural settings from the 10/66 Dementia in Developing Countries research program^18^ and a previous prospective study of African-Caribbean elderly adults in London^19^ and assessed the domains of global function (Community Screening Instrument for Dementia (CSID) cognitive assessment^20^), immediate and delayed verbal recall (Consortium to Establish a Registry for Alzheimer’s Disease 10-word list learning task^21^), verbal fluency (animal naming^22^), attention and mental flexibility (Color Trailmaking tests^23^), attention and immediate recall (forward and backward digit span^24^), and delayed visual recall (WHO gnostic assessments^25^). To accommodate South Asian participants who were not confident in English, a bilingual psychiatric nurse with expertise in dementia carefully translated the test instruments into Punjabi, with independent back-translation and review by a panel of clinicians with experience working closely with older people with dementia of South Asian ethnicity to ensure that they were as culturally fair as possible. A bilingual interviewer administered these assessments.

A composite measure of cognitive impairment was derived from this battery by standardizing individual test scores into z-scores (taking into account the distribution for the given ethnic group, subtracting the group mean from individual scores, and dividing each by the group standard deviation). These z-scores were then averaged for each participant to create a single score, an exploratory principal components analysis having previously established that there was a single-factor solution for each ethnic group in (data not shown). Stratifying the sample according to ethnicity, cognitive impairment as a binary outcome was defined according to a cutoff as close as possible to the 10th percentile for the group distribution. These composite variables for each of the three ethnic groups were combined to create a single variable for the sample as a whole (defining the 10% most cognitively impaired participants based on the distribution of scores within their ethnic group). For the 15.8% participants who did not have a complete set of cognitive data, average z-scores were calculated using only the tests they had completed, provided they had completed at least four, including the CSID. Impairment was defined using identical 10th percentile cutoffs established when those who had the complete set of cognitive outcomes were identified as impaired or not impaired. This was to take into account ethnic group differences in cognition as much as possible. Finally, test-specific cognitive impairment variables were defined for secondary analyses, based on cutoffs closest to 10th percentiles, considering the distributions within each ethnic group and categorizing participants accordingly.

### Statistical Analysis

Primary independent variables consisted of SBP and DBP. PP was calculated as SBP minus DBP and MAP was calculated as DBP + 1/3 PP. These variables and mean SBP and DBP at the three time points were divided into quintiles for initial analyses, and associations were investigated with cognitive impairment as the principal dependent variable. Multivariable analyses were performed using logistic regression, entering BP measures as continuous variables and then introducing a quadratic term. Crude associations were then adjusted for demographic factors (baseline age, interval between baseline and follow-up examinations, sex, education, and ethnicity), followed by a multivariable model with all covariates entered. Interactions between BP and ethnicity were tested using likelihood ratios in the full multivariable model. Because AER data were available only for a subsample, the analyzed sample was restricted to those with full data before investigating the effect of adjusting for this variable. Multivariable analyses of hypertensive treatment receipt as a binary variable were performed in the same way, including likelihood ratio tests for the interaction between treatment and ethnicity, as were those of ambulatory BP measurements (but without any interaction tests, because of the smaller sample size). Post hoc analyses were also performed stratifying according to age (above and below median) and hypertensive treatment. As mentioned, cognitive impairment had been reconstructed as a variable in those with incomplete data; sensitivity analyses restricted to the subsample with a full set of cognitive data were performed, but no substantial differences were found (data not shown). Secondary analyses were performed reassessing significant factors identified in primary analyses in relation to identically defined impairments for each of the individual component tests as separate outcomes. To investigate potential influences of attrition between baseline and follow-up examinations, a sensitivity analysis was performed using inverse probability weighting. These weights were calculated using logistic regression after estimation for the full baseline sample excluding those who subsequently died and entering all baseline covariates as predictors of participants’ presence or not in the final analyzed sample (successful follow-up with sufficient cognitive data for analysis). Primary analyses were then repeated, entering these postestimation probability coefficients as inverse weights (in effect, applying additional weighting to participants with characteristics that predicted attrition in the baseline sample).

## Results

Of 4,857 participants at baseline, 1,101 died, leaving 3,756. Of these, 306 did not respond, 241 had no UK address found, and 82 had emigrated. Of 3,433 traceable participants, 2,129 (62.0%) were directly followed up with 20 years later, and 1,484 of these had sufficient cognitive data to be included in the analysis (1,429 examined in clinic, 55 at home; 692 European, 551 South Asian, and 241 African Caribbean). Altogether, 172 (11.6%) participants with cognitive data were defined as having cognitive impairment. Compared to the baseline sample, those examined for cognitive function at follow-up were younger than the baseline sample (49.9 ± 6.2 and 52.4 ± 6.9, respectively) and had higher education (<10 years of education, 16.1% and 26.5%, respectively), but there was no substantial difference in sex distribution (female, 23.9% and 24.7%, respectively) or mean BP at baseline (121/78 and 125/79 mmHg, respectively). The range of intervals between baseline and follow-up examinations was 17 to 23 years (median 20 years).

**Figure 1:**
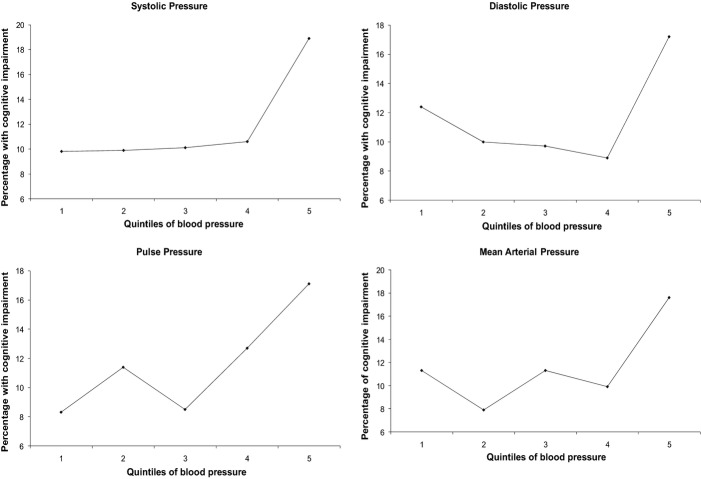
Prevalence (%) of cognitive impairment at follow-up according to quintile of systolic, diastolic, pulse, and mean arterial blood pressure at baseline.

As shown in Table 1, in unadjusted analyses, cognitive impairment was associated with the baseline characteristics of older age, female sex, lower education, diabetes mellitus, microalbuminuria, and higher BMI. Cognitive impairment was also associated with hypertension treatment at baseline, and significant heterogeneity was seen across quintiles of SBP and DBP, with a significant linear trend for SBP, PP, and MAP but not DBP. In terms of ambulatory BP in the subsample of 117 participants in whom these measurements were taken, there was a significant linear association between evening and nighttime ambulatory SBP and DBP and cognitive impairment. Figure 1 illustrates the relationship between BP quintiles at baseline and cognitive impairment at follow-up. Risk of cognitive impairment increased monotonically across the SBP and PP distributions, but the associations with DBP and MAP were U-shaped.

**Table:** Baseline Exposure and Unadjusted Associations with Cognitive Impairment at Follow-Up

Midlife Exposure	N	Cognitive Impairment at Follow-Up,%	Chi-Square (Degrees of Freedom), *P*-Value
Age			
40–49	755	6.1	45.33 (1), <.001
50–67	729	17.3	
Sex			
Male	1,129	10.5	5.08 (1), .02
Female	355	14.9	
Duration of education, years			
<10	234	29.1	97.84 (1), <.001
≥10	1,220	7.2	
Diabetes mellitus			
Absent	1,359	10.9	7.71 (1), .005
Present	125	19.2	
Microalbuminuria			
Absent	889	10.9	17.09 (1), <.001
Present	40	32.5	
Cardiovascular disease			
Absent	1,430	11.4	1.41 (1), .23
Present	54	16.7	
Body mass index, kg/m^2^			
<30.0	1,308	10.9	5.95 (1), .01
≥30.0	175	17.1	
Alcohol frequency per week			
<1	682	13.3	2.73 (1), .10
1–2	440	8.9	
≥Almost daily	349	10.6	
Smoking			
Never	876	12.0	0.41 (2), .81
Previous	264	11.0	
Current	341	10.9	
Hypertension treatment			
Present	129	21.7	14.11 (1), <.001
Absent	1,355	10.6	
SBP, mmHg			
82–108	306	9.8	8.19 (1), .004
109–116	324	9.9	
117–123	277	10.1	
124–134	302	10.6	
135–197	275	18.2	
DBP, mmHg			
39–70	348	12.4	1.60 (1), .21
71–75	281	10.0	
76–80	300	9.7	
81–87	282	8.9	
88–133	273	17.2	
Pulse pressure, mmHg			
17–34	302	8.3	10.35 (1), .001
35–39	307	11.4	
40–44	284	8.5	
45–51	299	12.7	
52–106	292	17.1	
Mean arterial pressure, mmHg			
61–82	302	11.3	6.24 (1), .01
83–88	292	7.9	
89–94	311	11.3	
95–101	284	9.9	
102–150	295	17.6	
Mean ambulatory SBP, mmHg, 9–11 a.m.			
88–116	24	4.2	1.57 (1), .21
117–129	23	13.0	
130–137	24	12.5	
138–150	23	13.0	
151–182	23	17.4	
Mean ambulatory DBP, mmHg, 9–11 a.m.			
51–72	24	12.5	0.81 (1), .37
73–81	24	4.2	
82–88	23	8.7	
89–99	24	20.8	
100–131	22	13.6	
Mean ambulatory SBP, mmHg, 5–7 p.m.			
79–113	32	0	12.48 (1), <.001
114–124	29	6.9	
125–134	32	6.3	
135–144	29	27.6	
145–195	30	23.3	
Mean ambulatory DBP, mmHg, 5–7 p.m.			
43–73	33	6.1	8.98 (1), .003
74–81	28	0	
82–88	31	9.7	
88–95	30	23.3	
95–125	30	23.3	
Mean ambulatory SBP, mmHg, 3–5 a.m.			
68–95	30	6.7	4.84 (1), .03
96–105	28	7.1	
106–115	30	10.0	
116–125	29	13.8	
126–178	28	25.0	
Mean ambulatory DBP, mmHg, 3–5 a.m.			
41–60	31	12.9	2.02 (1), .16
61–67	32	3.1	
68–74	26	3.9	
75–81	27	29.6	
82–105	29	13.8	

SBP = systolic blood pressure; DBP = diastolic blood pressure.

From analyses summarized in Table 2, the linear associations between SBP, PP, and cognitive impairment were attenuated after adjustment for demographic factors, although subsequent adjustment for other covariates had little further effect. An interaction term with ethnicity fell below statistical significance for SBP (likelihood ratio test chi-square (χ^2^) = 5.74, *P* = .06) and for PP (χ^2^ = 3.39, *P* = .18). Logistic regression analyses of DBP and MAP showed significant nonlinear relationships with cognitive impairment, with linear and quadratic terms remaining significant in the fully adjusted models and little changed after further adjustment for AER. No interactions with ethnicity were found for either variable (DBP, χ^2^ = .65, *P* = .72; MAP, χ^2^ = 1.84, *P* = .40). With post hoc stratification according to baseline age, the associations between DBP and MAP and cognitive impairment were strongest in the older half of the sample (50–67; for DBP, fully adjusted odds ratio (OR) = 0.06, 95% confidence interval (CI) = 0.01–0.41 linear, OR = 1.20, 95% CI = 1.07–1.34 quadratic; for MAP: OR = 0.08, 95% CI = 0.01–0.57 linear, OR = 1.14, 95% CI = 1.04–1.26 quadratic) and were not significant in the younger half (40–49; for DBP: OR = 0.33, 95% CI = 0.05–2.25 linear, OR = 1.08, 95% CI = 0.96–1.21 quadratic; for MAP: OR = 0.22, 95% CI = 0.03–1.89 linear, OR = 1.09, 95% CI = 0.98–1.21 quadratic). The lower DBP component of the nonlinear association was stronger in those who were not receiving antihypertensive treatment at baseline (linear term OR = 0.21, 95% CI = 0.05–0.80; quadratic term OR = 1.11, 95% CI = 1.02–1.20) than in those receiving antihypertensive treatment (linear term OR = 0.01, 95% CI = 0.00–5.66; quadratic term OR = 1.43, 95% CI = 0.96–2.14).

**Table:** Logistic Regression Analyses for Cognitive Impairment per 10-mmHg Increment in Each Listed Blood Pressure (BP) Measurement

			Diastolic BP			Mean Arterial Pressure	
Logistic Regression Model (Covariates Included)	N	Systolic BP	Linear Term	Quadratic Term	Pulse Pressure	Linear Term	Quadratic Term
				Odds Ratio (95% Confidence Interval), *P*-Value			
Unadjusted	1,484	1.20 (1.09–1.32), <.001	0.31 (0.10–0.98), .047	1.09 (1.01–1.17), .02	1.22 (1.06–1.40), .004	0.34 (0.10–1.18), .09	1.07 (1.00–1.14), .04
1 (demographic adjustments)	1,454	1.07 (0.96–1.19), .24	0.18 (0.05–0.65), .008	1.12 (1.04–1.21), .003	0.96 (0.81–1.13), .62	0.17 (0.04–0.63), .008	1.10 (1.03–1.18), .004
2 (fully adjusted)	1,431	1.06 (0.95–1.20), .30	0.15 (0.04–0.56), .004	1.13 (1.05–1.22), .002	0.95 (0.80–1.12), .51	0.13 (0.03–0.52), .004	1.12 (1.04–1.20), .002
2 in participants with AER data	895	1.11 (0.95–1.29), .19	0.10 (0.02–0.54), .007	1.16 (1.05–1.28), .003	0.97 (0.79–1.21), .81	0.07 (0.01–0.39), .003	1.16 (1.06–1.26), .001
2 plus AER	895	1.09 (0.94–1.27), .26	0.10 (0.02–0.52), .006	1.16 (1.06–1.28), .003	0.97 (0.78–1.20), .75	0.06 (0.01–0.38), .002	1.16 (1.06–1.27), .001

Model 1: adjusted for age at follow-up, interval between baseline and follow-up, sex, education, ethnicity.

Model 2: adjusted for Model 1 covariates plus smoking, alcohol intake, total cholesterol level, cardiovascular disease, obesity, diabetes mellitus.

AER = albumin excretion rate.

Table 3 summarizes analyses of the association between hypertensive treatment at baseline and cognitive impairment at follow-up. In summary, although attenuated by adjusting for age and sex, this association persisted after adjusting for covariates and after further adjustment for AER. No significant interaction with ethnicity was found (χ^2^ = .11, *P* = .94), and post hoc adjustment for DBP and MAP made little difference (data not shown).

**Table:** Logistic Regression Analyses for Cognitive Impairment at Follow-Up in Participants Undergoing Hypertensive Treatment at Baseline Compared with All Other Participants

Model	N	Odds Ratio (95% Confidence Interval), *P*-Value
Unadjusted	1,484	2.33 (1.48–3.67), <.001
1 (demographic adjustments)	1,454	1.71(1.02–2.86), .04
2 (fully adjusted)	1,431	1.92 (1.13–3.25), .01
2 in participants with AER data	895	2.15 (1.13–4.09), .02
2 plus AER	895	2.11 (1.10–4.03), .02

Model 1: adjusted for age at follow-up, interval between baseline and follow-up, sex, education, ethnicity.

Model 2: adjusted for Model 1 covariates plus smoking, alcohol intake, total cholesterol level, cardiovascular disease, obesity, diabetes mellitus.

AER = albumin excretion rate.

Sensitivity analyses using inverse probability weighting (to take attrition between baseline and follow-up interviews into account) revealed similar findings. In fully adjusted models (Model 2 in Tables 2 and 3), ORs for cognitive impairment were as follows: SBP, OR = 1.08 (95% CI = 0.95–1.23), DBP linear, OR = 0.12 (95% CI = 0.03–0.54), DBP quadratic, OR = 1.15 (95% CI = 1.05–1.26), PP, OR = 0.93 (95% CI = 0.79–1.10), MAP linear, OR = 0.10 (95% CI = 0.02–0.57), MAP quadratic, OR = 1.13 (95% CI = 1.04–1.24), antihypertensive treatment, OR = 2.00 (95% CI = 1.16–3.42).

Table 4 displays logistic regression analyses from the subsample that underwent ambulatory BP monitoring. No associations were found with later cognitive function for average morning or nighttime BP, but there were linear associations between evening SBP and DBP and likelihood of cognitive impairment that were largely unaltered after individual adjustments for covariates, although evening SBP was no longer significant in the fully adjusted model. Further adjustments for resting DBP or MAP or for antihypertensive treatment had little effect on the association between evening DBP and cognitive impairment (data not shown).

**Table:** Logistic Regression Analyses Representing for Cognitive Impairment per 10-mmHg Increment in Each Ambulatory Blood Pressure Measurement in the Subsample (117 African-Caribbean and European Participants) in Which This Was Measured

	Morning (9–11 a.m.)			Evening (5–7 p.m.)			Night (3–5 a.m.)		
		SBP	DBP		SBP	DBP		SBP	DBP
Model	n	OR (95% CI), *P*-Value		n	OR (95% CI), *P*-Value		n	OR (95% CI), *P*-Value	
Unadjusted	117	1.19 (0.91–1.56), .21	1.09 (0.75–1.59), .65	152	1.52 (1.16–1.97), .002	1.67 (1.13–2.46), .01	145	1.31 (1.00–1.72), .049	1.40 (0.95–2.08), .09
1 (demographic adjustments)	117	1.05 (0.76–1.44), .77	0.92 (0.60–1.40), .69	152	1.38 (1.02–1.85), .04	1.94 (1.18–3.20), .009	145	1.22 (0.90–1.65), .20	1.35 (0.85–2.14), .20
2 (fully adjusted)	112	1.02 (0.66–1.58), .91	0.91 (0.45–1.81), .78	145	1.29 (0.88–1.87), .19	2.97 (1.20–7.33), .02	139	1.26 (0.79–2.00), .33	1.76 (0.90–3.42), .10
2 in participants with AER data	91	1.24 (0.69–2.22), .47	1.37 (0.57–3.27), .48	112	1.93 (1.05–3.54), .03	3.19 (1.13–9.01), .03	108	1.04 (0.60–1.79), .89	1.92 (0.87–4.24), .10
2 plus AER	91	1.02 (0.53–1.98), .95	1.13 (0.44–2.88), .80	112	1.71 (0.89–3.26), .11	2.87 (0.88–9.37), .08	108	0.82 (0.44–1.55), .54	1.64 (0.73–3.71), .23

Model 1: adjusted for age at follow-up, interval between baseline and follow-up, sex, education, ethnicity.

Model 2: adjusted for Model 1 covariates plus smoking, alcohol intake, total cholesterol level, cardiovascular disease, obesity, diabetes mellitus.

OR = odds ratio; CI = confidence interval; SBP = systolic blood pressure; DBP = diastolic blood pressure; AER = albumin excretion rate.

Table S1 displays logistic regression analyses of cognitive impairment according to individual tests as dependent variables (with impairment on each defined on the basis of a score within the lowest 10% of the distribution within each ethnic group). In summary, most of the associations were strongest for verbal fluency as an outcome, particularly the U-shaped relationships with DBP and MAP. Antihypertensive treatment was most strongly associated with impaired immediate verbal and visual recall.

## Discussion

Using data from a long-running multiethnic cohort study, associations between current cognitive impairment and markers of hypertensive disease 20 years earlier were investigated. In summary, an independent U-shaped association was found with resting DBP and MAP levels (cognitive impairment associated with high and low levels) and independent associations with midlife use of antihypertensive agents and with higher evening DBP in those who underwent ambulatory BP monitoring. The U-shaped associations with resting DBP and MAP were more prominent in older than younger participants. Considering potential underlying mechanisms, resting PP (as a marker of arterial stiffness) showed little evidence of association, and AER (as a measure of small vessel disease) did not have any substantial influence on the associations of interest. The associations did not vary substantially between ethnic groups.

Strengths of the study were the 20-year interval between exposure and outcome and comprehensive assessment of BP at baseline, allowing examination of a variety of different features of hypertension as exposures. Measures of ambulatory BP were only made in European and African-Caribbean participants and were therefore not representative of the sample at baseline, although they are unlikely to be biased with respect to their associations with cognitive function 20 years later. Cognitive assessment was comprehensive and used procedures that had been specifically designed for their cross-cultural applicability, in particular, the CSID, which has received substantial international evaluation.^17^ Weaknesses of this study include limited statistical power in some respects, particularly for stratified analyses. The sample examined should also be considered to be relatively healthy survivors constituting a small proportion of the original participants, although those with and without cognitive impairment were drawn from the same source (survivors from the original cohort), and a sensitivity analysis using inverse probability weighting to account for differential attrition did not meaningfully alter findings. Hypertension and cognitive impairment are predictors of mortality, so survival effects would more likely have obscured than exaggerated associations. A large number of covariates were considered, although residual confounding cannot be excluded absolutely. There was also no baseline information on cognitive function and therefore no means of measuring cognitive decline. Finally, no attempt was made to test mediating pathways, so measures of health status at follow-up were not considered as covariates.

The most consistent findings relating BP levels to cognitive impairment in later life have arisen from samples followed for at least 10 years. The findings of the current study that hypertension was associated with cognitive impairment 20 years later are in keeping with this literature, although other findings of worse late-life cognitive function associated with progressively higher midlife DBP 3 differ from those of the current study, in which a U-shaped relationship was found. Other studies of cognitive function with BP ascertainment at least 10 years earlier have reported associations with high midlife SBP (≥160 vs <110 mmHg) in men,^26^ with progressively higher midlife SBP in people who had not received antihypertensive treatment,^2^ with persistently high SBP (≥140 mmHg) over a 38-year period, and with high SBP (≥160 vs <140 mmHg).^4^ In the Framingham Offspring cohort study, midlife hypertension was specifically associated with worse late-life executive function in addition to progression of white matter hyperintensities on magnetic resonance imaging.^27^ Findings from studies with dementia as an outcome include associations between high midlife SBP and DBP (≥160/95 mmHg) in men and dementia and AD, although only in those not previously taking antihypertensive agents,^28^ and an association between midlife high SBP (≥160 vs <140 mmHg) but not high DBP and AD.^29^ Therefore, although hypertension is implicated in late-life cognitive impairment, there is still a substantial level of controversy around what components of BP are most important and in what subgroups.

U-shaped associations between BP and cognitive impairment are not uncommonly found, and there is ample evidence of an exaggerated decline in BP (particularly SBP) preceding the clinical onset of dementia.^1^ It is less usual to find a U-shaped relationship over a 20-year interval, as described here for DBP and MAP. The greater risk in those with lower pressures is unlikely to represent a secondary effect of neurodegeneration (often assumed to underlie more-contemporaneous associations between lower BP and cognitive impairment). Low BP might reflect atherosclerotic disease, or it might be a longstanding feature by which the individual is rendered vulnerable to episodes of hypoperfusion. It might also be a marker of a more-general frailty syndrome that predisposes to later cognitive impairment. The apparently stronger associations in older than younger participants at baseline supports this, although it requires replication. The association with lower DBP appeared stronger in people not taking antihypertensive agents and was therefore not likely to be iatrogenic in nature. Finally, the relationships were stronger for verbal fluency than memory. Although Type 1 statistical error is possible because this association was one of several exploratory analyses, this stronger association with verbal fluency may be consistent with subcortical damage secondary to cerebrovascular disease causing impairment in executive function rather than memory.

Fewer data have been published on midlife PP than on SDB and DBP as an exposure for late-life cognitive impairment. The Women’s Health Study found a five times greater likelihood of impairment on a measure of executive function in women with PP of 84 mmHg or greater than in women aged 76 to 80 with PP less than 68 mmHg and twice the incidence of impairment on immediate recall in participants aged 70 to 75 with PP of 71 mmHg than in those with PP less than 68 mmHg.^10^ The Kungsholmen study found a U-shaped relationship between PP and dementia in women aged 75 and older.^8^ These studies measured a potentially much later stage of vascular pathology in older age groups, but considerably less is known about the role of midlife PP in relation to later cognitive impairment. SBP is reported to increase with age, whereas DBP increases up to aged 50 to 59 and then begins to decline so that PP increases in later life.^30^ This may explain why the current study found little evidence of midlife PP as an independent predictor of later cognitive impairment. Similarly, the Honolulu Asia Aging study found no association between PP in midlife and incidence of dementia in later life.^31^ One possible explanation is that PP is not important in the etiology of cognitive impairment, a second is that this only becomes important closer to the development of the outcome, and a third is that people with higher PP in midlife who survive to late life are healthy in other respects, masking associations with later cognition. Midlife AER did not appear to play a substantial role in the association between BP and cognition, even though baseline microalbuminuria was associated with cognitive impairment.

The findings on ambulatory BP are novel in terms of the length of follow-up between exposure and outcome. Evening BP was more strongly associated with cognitive impairment than morning and nocturnal measurements. Previous research^32^ has found high nocturnal SBP in older adults (mean age 71),^33^ lack of a nocturnal dip,^34^ and exaggerated variability in very elderly adults 35 to be associated with cognitive impairment and dementia. The cohort in the current study was younger at the time of ambulatory measurement, and although some evidence of an association between nocturnal BP and later-life cognitive impairment was found, this disappeared after adjustment for age and sex. Mean resting BP might underestimate an additional influence of BP variability, although whether there are particular high-sensitivity periods during the day or night remains to be established. An important consideration is that the timing of bed rest influenced evening BP, and other factors might have confounded this association, which would require further investigation.

If midlife BP is associated with later-life cognitive impairment and subsequent dementia, management of BP in midlife could have important public health implications in terms of prevention. Several trials have been conducted of BP lowering and cognitive outcomes,^36^–^41^ although only the Systolic Hypertension in Europe Study found a significant primary effect on risk of dementia, with a reduction in incidence from 7.7 to 3.8 per 1,000 person-years,^37^ findings that persisted in open-label follow-up.^42^ In the current study sample, people receiving antihypertensive treatment at baseline were more likely to have cognitive impairment 20 years later, although antihypertensive use at baseline reflects chronic exposure to hypertension as well as its treatment. Overall, these findings may have important clinical implications in terms of the importance of detection and control of high BP in reducing the risk of cognitive impairment later in life.

## SUPPORTING INFORMATION

**Table S1.** Adjusted* odds ratios (95% CI) from logistic regression analyses for cognitive impairment (lowest 10% performance within ethnic group) on individual cognitive tests per 10 mmHg increment in each listed blood pressure measurement. Please note: Wiley-Blackwell is not responsible for the content, accuracy, errors, or functionality of any supporting materials supplied by the authors. Any queries (other than missing material) should be directed to the corresponding author for the article.
