# Supplementary material for: Midlife Hypertensive Status and Cognitive Function 20 Years Later: The Southall and Brent Revisited Study
Source: J Am Geriatr Soc. 2013 Aug 26;61(9):1489–98. doi: 10.1111/jgs.12416 (PMC3902992; doi:10.1111/jgs.12416)
Supplement: Supplementary file 1 — Table S1. Adjusted* odds ratios (95% CI) from logistic regression analyses for cognitive impairment (lowest 10% performance within ethnic group) on individual cognitive tests per 10 mmHg increment in each listed blood pressure measurement. [file jgs0061-1489-sd1.docx]

**Supplemental Material**

| **Supplemental Table: Adjusted* odds ratios (95%CI) from logistic regression analyses for cognitive impairment (lowest 10% performance within ethnic group) on individual cognitive tests per 10mmHg increment in each listed blood pressure measurement** | | | | | | | |
| --- | --- | --- | --- | --- | --- | --- | --- |
| **Logistic regression** | **N** | **Hypertensive treatment** | **Diastolic pressure** | | **Mean arterial pressure** | |  |
|  |  |  | **Linear term** | **Quadratic term** | **Linear term** | **Quadratic term** |  |
| Immediate word list recall | 1430 | **1.76(1.02,3.04)**  P=0.041 | 1.16(0.23,5.77)  P=0.859 | 1.00(0.90,1.10)  P=0.941 | 1.03(0.21,5.13)  P=0.974 | 1.00(0.93,1.09)  P=0.925 |  |
| Delayed word list recall | 1431 | 0.77(0.42,1.40)  P=0.385 | 0.42(0.12,1.52)  P=0.185 | 1.05(0.97,1.14)  P=0.206 | 0.48(0.12,1.96)  P=0.307 | 1.04(0.96,1.11)  P=0.337 |  |
| Visual recognition | 1262 | **2.01(1.45,3.50)**  P=0.014 | 0.70(0.13,3.71)  P=0.673 | 1.03(0.93,1.14)  P=0.591 | 0.45(0.09,2.23)  P=0.327 | 1.05(0.96,1.13)  P=0.284 |  |
| Digit span forward | 1426 | 1.41(0.81,2.46)  P=0.224 | 1.02(0.21,4.82)  P=0.983 | 1.01(0.92,1.11)  P=0.874 | 1.06(0.22,5.27)  P=0.939 | 1.00(0.93,1.09)  P=0.929 |  |
| Digit span backwards | 1420 | 1.50(0.83,2.72)  P=0.181 | 0.43(0.08,2.36)  P=0.332 | 1.05(0.95,1.16)  P=0.333 | 0.28(0.05,1.50)  P=0.138 | 1.07(0.98,1.16)  P=0.133 |  |
| Verbal fluency (animal naming) | 1431 | 1.74(0.99,3.07)  P=0.055 | **0.25(0.07,0.90)**  P=0.034 | **1.10(1.02,1.19)**  P=0.013 | **0.15(0.04,0.57)**  P=0.005 | **1.11(1.04,1.19)**  P=0.002 |  |
| Trail A time | 1393 | 1.45(0.80,2.63)  P=0.216 | 0.40(0.10,1.56)  P=0.189 | 1.07(0.99,1.16)  P=0.102 | 0.33(0.08,1.35)  P=0.124 | **1.07(1.00,1.15)**  P=0.067 |  |
| Trail B time | 1375 | 1.21(0.65,2.24)  P=0.549 | 0.47(0.12,1.88)  P=0.284 | 1.05(0.97,1.15)  P=0.215 | 0.38(0.09,1.65)  P=0.199 | 1.06(0.98,1.14)  P=0.149 |  |
| Global function (CSID) | 1431 | 1.71(0.96,3.02)  P=0.067 | 0.48(0.12,1.85)  P=0.284 | 1.05(0.97,1.14)  P=0.235 | 0.37(0.09,1.54)  P=0.173 | 1.06(0.98,1.13)  P=0.137 |  |

*Adjusted for baseline age, follow-up interval, sex, education, ethnicity, smoking, alcohol intake, total cholesterol level, cardiovascular disease, obesity, diabetes

Significant (p<0.05) odds ratios displayed in bold font.
